# Management of Beta-Lactam Antibiotics Allergy: A Real-Life Study

**DOI:** 10.3389/falgy.2022.853587

**Published:** 2022-04-08

**Authors:** Sarah Iuliano, Laurence Senn, Laura Moi, Yannick D. Muller, Camillo Ribi, Guillaume Buss, Denis Comte

**Affiliations:** ^1^Service of Immunology and Allergy, Lausanne University Hospital, Lausanne, Switzerland; ^2^Faculty of Biology and Medicine, University of Lausanne, Lausanne, Switzerland; ^3^Service of Hospital Preventive Medicine, Lausanne University Hospital, Lausanne, Switzerland

**Keywords:** allergy, beta-lactam, penicillin, cephalosporin, carbapenem

## Abstract

Beta-lactam allergy is a common problem in everyday medical practice and is recognized as a major public health issue. Carrying this label frequently leads to the avoidance of all beta-lactam antibiotics, favoring the use of other less preferred classes of antibiotics, that are more expensive and associated with more side effects and increased antimicrobial resistance. Therefore, delabeling a beta-lactam allergy is part of antimicrobial stewardship programs. Herein, we retrospectively examined the clinical records of 576 patients who were referred to our center for a label of allergy to beta-lactam antibiotics and were systematically investigated following a standardized algorithm. Our main aim was to evaluate the frequency of confirmed immediate- and delayed-type allergy to commonly prescribed subclasses of beta-lactam antibiotics (penicillin and cephalosporin), as well as the negative predictive value (NPV) and the sensitivity of skin tests. Our secondary aims were to examine the safety of beta-lactam skin testing and drug challenge. We identified that 260 patients reported a history of immediate reactions, 131 of delayed reactions, and 114 of unknown timing or mechanism of reactions. Following assessment and testing, 86 (18.3%) patients had a confirmed allergy to any beta-lactam antibiotics; 63 (13.4%) with an immediate- and 23 (4.9%) with a delayed-type reaction. Most frequently identified confirmed allergy was to penicillins (65 patients), followed by cephalosporins (21 patients). When immediate-type reactions were examined, NPV of skin tests were 96.3% and 100% for penicillins and cephalosporins, respectively. When delayed reactions were considered, NPV were 91.9 and 87.5% for penicillins and cephalosporins, respectively. Evaluation of the safety of skin tests according to the standardized procedure showed that systemic allergic reactions occurred in only 0.7% of skin tests and in 3.1% of drug challenges. Overall, our data indicate that only 18.3% of patients with a beta-lactam allergy label have a confirmed allergy and non-allergic patients can be safely delabeled through allergic workup based on skin tests and drug challenge. This approach supports the policy of saving second-line antibiotics through a standardized allergy workup.

## Introduction

Beta-lactam allergy is a common problem in everyday medical practice and is recognized as a major public health issue (1). Studies indicate that ~10% (2) of the general population and 15% of the hospital population (3) are labeled with a penicillin allergy. This label is often misleading because contemporary publications indicate that <10% of these patients are truly allergic after an oral drug challenge (4). Carrying this label frequently leads to the avoidance of all beta-lactam antibiotics, favoring the use of other less preferred classes of antibiotics, that are more expensive and associated with more side effects and increased antimicrobial resistance (5). Delabeling a beta-lactam allergy is part of antimicrobial stewardship programs.

**Figure 1:**
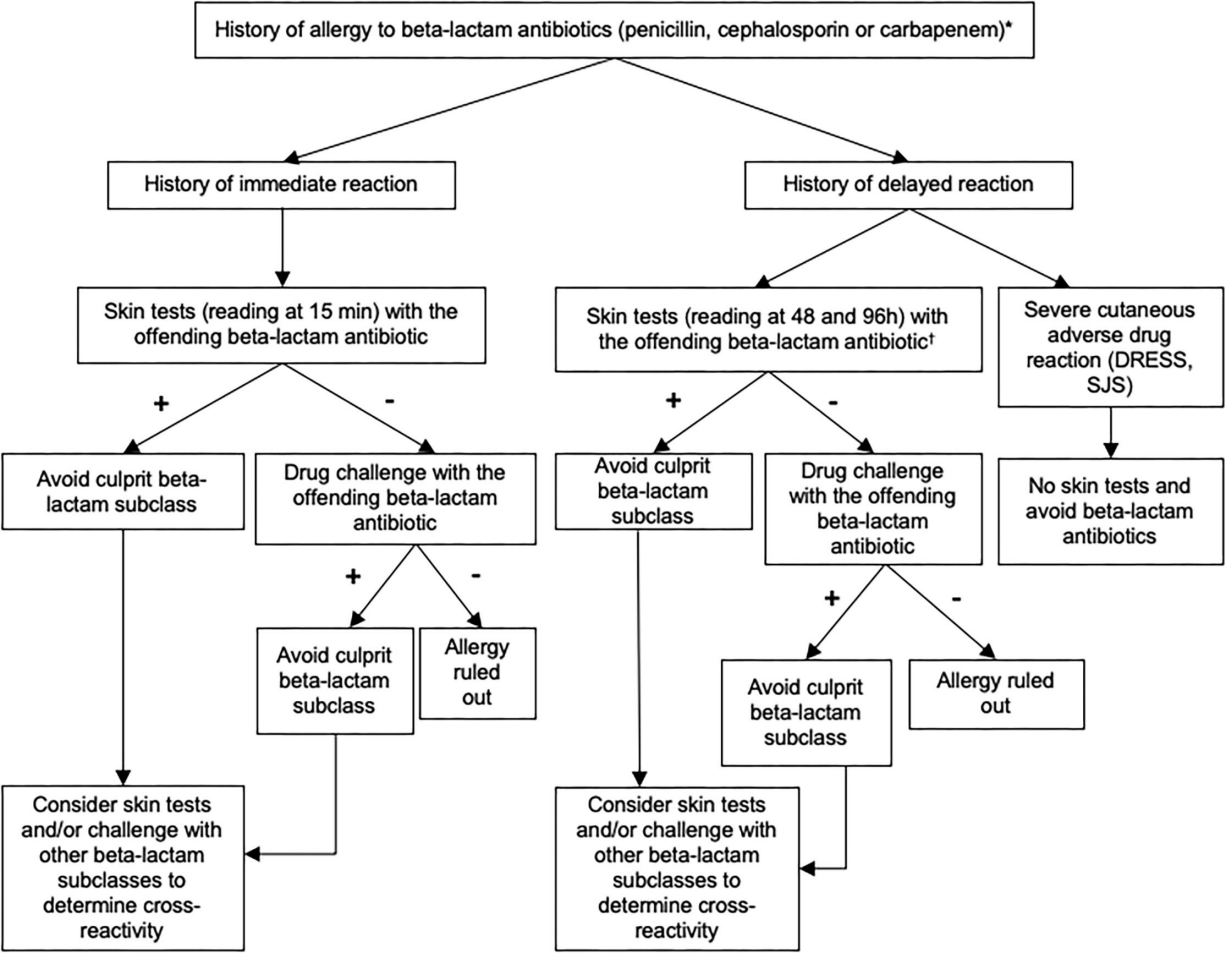
Recommended algorithm for assessing patients with a suspected allergy to beta-lactam antibiotics. These recommendations can be adapted according to the judgment of the allergist physician. Skin test reading at 15 min for immediate-type reaction and at 48 and 96 h for delayed-type reaction. Standard tests (prick skin puncture and intradermal testing): penicilloyl-polylysine (PPL), minor determinant mixture (MDM), benzylpenicillin, amoxicillin. Optional tests (according to exposure and medical history): amoxicillin/clavulanate, piperacillin/tazobactam, flucloxacillin, cefuroxime, ceftriaxone, cefazolin, ceftazidime, cefpodoxime, cefepime, cefixime, meropenem, imipenem/cilastatin, ertapenem. *If the phenotype of the reaction was unknown, skin testing should include both immediate and delayed readings. In some cases, a direct drug challenge (i.e., without prior skin testing) was performed on the basis of the physician's assessment because the risk of a reaction was highly unlikely.^†^Skin tests for delayed-type allergy should not be performed in patients with a history of severe exfoliative reaction to beta-lactam antibiotics. DRESS, Drug Reaction with Eosinophilia and Systemic Symptoms; SJS, Stevens-Johnson syndrome.

Since 2011, a decisional algorithm has been set up at the Centre Hospitalier Universitaire Vaudois (CHUV) to optimize the management of patients labeled with a beta-lactam allergy (Figure 1). This algorithm is based on clinical history, skin testing [prick tests and intradermal reactions (IDR)] and drug challenges (oral, intravenous, or intramuscular) (6) and allows to identify patients who are truly allergic and to delabel the allergy to beta-lactam in whom it is not proven.

In this study, we performed a retrospective analysis of patients who were referred to our university allergy clinic with a history of allergy to beta-lactam antibiotics. We assessed the true frequency of immediate and delayed allergies to commonly prescribed subclasses of beta-lactam antibiotics (including penicillins and cephalosporins) and the negative predictive value (NPV) and the sensitivity of skin tests. Our secondary aims were to examine the safety of beta-lactam skin testing and drug challenge.

## Methods

Medical files of all consecutive patients seen at the outpatient clinic between January 1st 2011 and December 31st 2018 were retrospectively screened for the following keywords: “*beta-lactam, penicillin, cephalosporin, carbapenem, amoxicillin, amoxicillin/clavulanate, flucloxacillin, piperacillin-tazobactam, cefuroxime, cefpodoxime, cefazolin, cefepime, imipenem-cilastatin, ertapenem, meropenem, penicilloyl-polylysine (PPL), minor determinant mixture (MDM).”* Any patients with a beta-lactam antibiotic listed in their electronic files were included even if there was no notion of an allergy (for example, the absence of an allergy to beta-lactam antibiotics was often listed in the medical file and was detected by the keyword screening). Further examination of the medical records that met the above criteria identified patients who were referred to investigate a history of an allergy to beta-lactam antibiotics. Of note, patients with an allergy to carbapenems were removed from the analysis due to their limited number.

The following data were collected for patients referred for a history of an allergy to beta-lactam antibiotics: age, gender, history of atopy and autoimmune disease, history of reaction to beta-lactam antibiotics (type, grade, date, antibiotic molecule involved, and medical management of the reaction), beta-lactam skin test results, beta-lactam drug challenge results, adverse reaction to skin tests and drug challenges.

Immediate reactions were defined as reactions that occurred up to 6 h but usually during the first hour after beta-lactam exposure and included the following clinical features: hives, angioedema, bronchospasm and/or hypotension (7). Severity of the reaction was graded from I to IV according to Müller's grading system (8) (in brief, grade I: hives; grade II: angioedema, gastrointestinal manifestations; grade III: bronchospasm, grade IV: hypotension). Delayed reactions were defined as reactions that occurred more than 6 h after drug exposure and featured maculopapular eruptions or more severe hypersensitivity reactions such as drug reaction with eosinophilia and systemic symptoms (DRESS), Stevens-Johnson syndrome (SJS), and toxic epidermal necrolysis (TEN). For reactions occurring between 1 and 6 h, we used the phenotype of the reaction at first to determine if it was an immediate or a delayed-type of reaction. Unknown timing or mechanism of reactions were defined by reactions for which information on timing or the mechanism of reaction after beta-lactam exposure were missing.

A diagnosis of confirmed allergy was based on a positive skin test and/or drug challenge. Skin testing workup is summarized in Supplementary Table 1. If the phenotype of the reaction was unknown, skin testing should include both immediate and delayed readings.

The performance of skin testing was evaluated in patients with results of both skin test and drug challenge, with distinction between immediate and delayed-type reactions. In rare instance, a direct drug challenge (i.e., without prior skin testing) was performed on the basis of the physician's assessment because the risk of a reaction was highly unlikely. The majority of drug challenges (*N* = 270) consisted of a single drug dose. A minority of drug challenges (*N* = 18) were realized on 3 days with a single drug dose per day according to the decision of the investigator.

We examined the safety of the current standardized approach to evaluate patients with a beta-lactam allergy label, which includes a thorough evaluation of the medical history, followed by skin tests and drug challenge.

The study was conducted in accordance with the Declaration of Helsinki. The project was approved by the Ethics Committee of the Canton of Vaud (CER-VD 2019-00337) that waived the need for informed consent allowing the inclusion of all patients except those who refused the use of their data. All data were anonymized before analysis. Data analysis was performed using Stata software version 16.1 for Windows (Stata Corp LCC).

## Results

Of the 2,320 patients screened for a history of allergy to beta-lactam, 576 met the inclusion criteria and were included in the analysis (Tables 1, 2). Mean age of the patients was 49.7 years (SD ± 17.7) and 65.3% were female. Eighty five patients reported more than one reaction (which included both multiple reactions to the same class of beta-lactam antibiotics and reactions to different classes of beta-lactam antibiotics). Patients were divided into three groups according to the reported type of reaction: 309 patients had a history of an immediate-type and 172 of a delayed-type reaction (Table 1). In 138 patients, the timing or mechanism of the reaction was unknown (Table 2). Twelve patients reported reactions to both penicillins and cephalosporins in the immediate-type reaction group, and 8 in the delayed-type reaction group. Twelve patients reported both immediate- and delayed-type reactions and were included in both groups.

**Table 1:** Characteristics of 436 patients with a history of immediate- and delayed-type reactions to beta-lactam antibiotics.

**History of immediate reaction**		**History of delayed reaction**	
Total number of patients, *n*	309	Total number of patients, *n*	172
Mean age (years) ± SD	51.2 ± 16.8	Mean age (years) ± SD	47.8 ± 18.9
Female (%)	63.1	Female (%)	66.5
Culprit beta-lactam, *n* (%)	All reaction (*n* = 346)	Culprit beta-lactam, *n* (%)	All reaction (*n* = 200)
**Penicillin**	**258 (76.1)**	**Penicillin**	**160 (84.2)**
Unspecified	113 (33.3)	Unspecified	31 (16.3)
Amoxicillin/clavulanate	100 (29.5)	Amoxicillin/clavulanate	82 (43.2)
Amoxicillin	32 (9.4)	Amoxicillin	30 (15.8)
Piperacillin/tazobactam	11 (3.3)	Piperacillin/tazobactam	11 (5.8)
Flucloxacillin	2 (0.6)	Flucloxacillin	5 (2.6)
		Ampicillin	1 (0.5)
**Cephalosporin**	**81 (23.9)**	**Cephalosporin**	**30 (15.8)**
Cefuroxime	51 (15.0)	Cefuroxime	14 (7.4)
Ceftriaxone	15 (4.4)	Ceftriaxone	7 (3.7)
Cefazolin	9 (2.7)	Cefepime	5 (2.6)
Ceftazidime	1 (0.3)	Cefazolin	2 (1.1)
Cefpodoxime	1 (0.3)	Ceftazidime	1 (0.5)
Cefepime	1 (0.3)	Cefpodoxime	1 (0.5)
Cefixime	1 (0.3)		
Unspecified	2 (0.6)		
Severity grading according to J.L. Müller Manifestations		Manifestations	
Grade IV (anaphylactic shock)	75 (22.1)	Benign skin rash^*^	181 (95.3)
Grade III (bronchospasm)	80 (23.6)	DRESS	3 (1.6)
Grade II (angioedema)	56 (16.5)	SJS	1 (0.5)
Grade I (generalized urticaria)	108 (31.9)	Unknown	5 (2.6)
Unspecified	20 (5.9)		

*DRESS, Drug Reaction with Eosinophilia and Systemic Symptoms; SJS, Stevens-Johnson syndrome*.

* *Maculopapular exanthema that did not require any treatment*.

**Table 2:** Management of patients with a suspected allergy to beta-lactam antibiotics and unknown timing or mechanism of reaction-type.

Unknown timing or mechanism of reaction			
Total number of patients, *n*			138
Mean age (years) ± SD			48.4 ± 18.1
Female (%)			68.8
Culprit beta-lactam, *n* (%)			All reaction (*n* = 144)
	**Penicillin**		**126 (87.5)**
	Unspecified		95 (66.0)
	Amoxicillin/clavulanate		18 (12.5)
	Amoxicillin		10 (7.0)
	Piperacillin/tazobactam		3 (2.1)
	**Cephalosporin**		**16 (11.1)**
	Cefuroxime		9 (6.3)
	Cefazolin		2 (1.4)
	Cefepime		1 (0.7)
	Ceftazidime		1 (0.7)
	Unspecified		3 (2.1)
	**Unspecified**		**2 (1.4)**
Manifestations			
	Benign skin rash^*^		64 (44.4)
	Skin rash and angioedema		13 (9.0)
	Angioedema		10 (7.0)
	Others (digestive symptoms)		4 (2.8)
	Unknown		47 (32.6)
	No reaction		6 (4.2)

* *Maculopapular exanthema that did not require any treatment*.

### Global Evaluation of Allergy to Beta-Lactam Antibiotics

The prevalence of confirmed allergies to beta-lactam antibiotics based on positive skin tests and drug challenges was 18.3% (86/471). This included 13.4% (63/471) with immediate-type allergies and 4.9% (23/471) with delayed-type allergies.

Of the 63 patients with a confirmed immediate-type allergy, 59 had positive skin tests and 4 a positive drug challenge. Of the 23 patients with a delayed hypersensitivity, 18 had a delayed positive skin test and 5 a positive drug challenge (Supplementary Tables 2A,B).

### Penicillin Allergy

**Figure 2:**
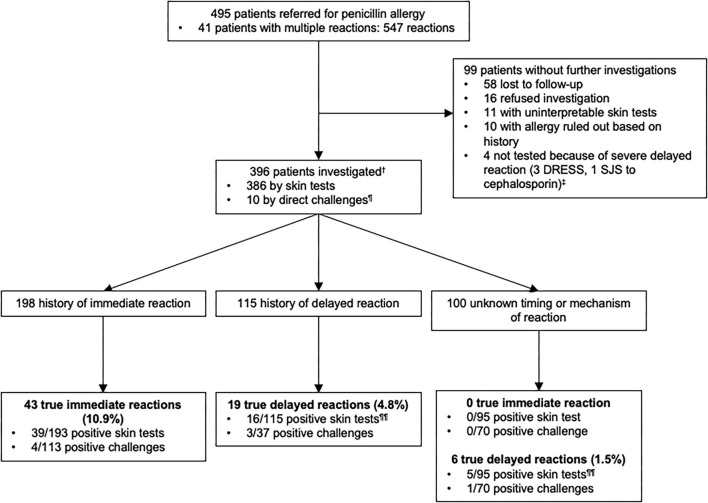
Outcomes of testing in 396 patients with a history of penicillin allergy.^†^Among 396 patients who were studied for a history of allergy to penicillin antibiotics, some of them had multiple reactions and were included in more than one group.^‡^1 patient reported a delayed-type reaction with a penicillin but did not underwent skin tests because he had a SJS with a cephalosporin. ^¶^5 patients had negative direct drug challenge without prior skin testing (5 direct for suspicion of an immediate-type reaction and 5 for reaction of unknown timing or mechanism of the reaction-type). Direct drug challenges were performed on the basis of the physician's assessment because the risk of a reaction was highly unlikely. ^¶¶^3 patients with a confirmed allergy, reported multiple allergic reactions to penicillin antibiotics and were included both in delayed and unknown timing or mechanism of the reaction groups according to the clinical description of the reactions. DRESS, Drug Reaction with Eosinophilia and Systemic Symptoms; SJS, Stevens-Johnson syndrome.

Of the 495 patients referred for a history of allergy to penicillins (Figure 2), 396 were investigated. One hundred and ninety eight patients were investigated for a history of immediate-type reaction to penicillins and because some patients had more than one reaction, this represents a total of 212 reactions. These included 12.7% (*N* = 27) stage IV reactions, 25.0% (*N* = 53) stage III, 55.7% (*N* = 118) stage I–II, and 6.6% (*N* = 14) unspecified according to Müller's classification of immediate-type allergic reaction. 10.9% (*N* = 43) out of 396 patients had a confirmed immediate-type allergy to penicillins.

One hundred and fifteen patients were investigated for a history of delayed-type reaction with a total of 122 reactions that included 99.2% (*N* = 121) benign cutaneous maculopapular eruptions. In 0.8% (*N* = 1), clinical characteristic of the reaction was not available. Only 4.8% (19/396) of the patients had a confirmed delayed-type allergic reaction to penicillins.

In the unknown timing or mechanism of the reaction group of patients (*N* = 100), 1.5% (*N* = 6) of the patients had a confirmed delayed-type allergy to penicillins. Of note, 3 patients with a confirmed allergy were included both in delayed and unknown timing or mechanism of reactions groups according to the clinical description of the reaction. Thus, we only have a total of 22 positive patients instead of 25 for a confirmed delayed-type allergy.

Our data indicated that the prevalence of confirmed penicillins allergies was 16.4% (65/396) overall, including 10.9% (43/396) immediate-type and 5.6% (22/396) delayed-type reactions.

### Cephalosporin Allergy

**Figure 3:**
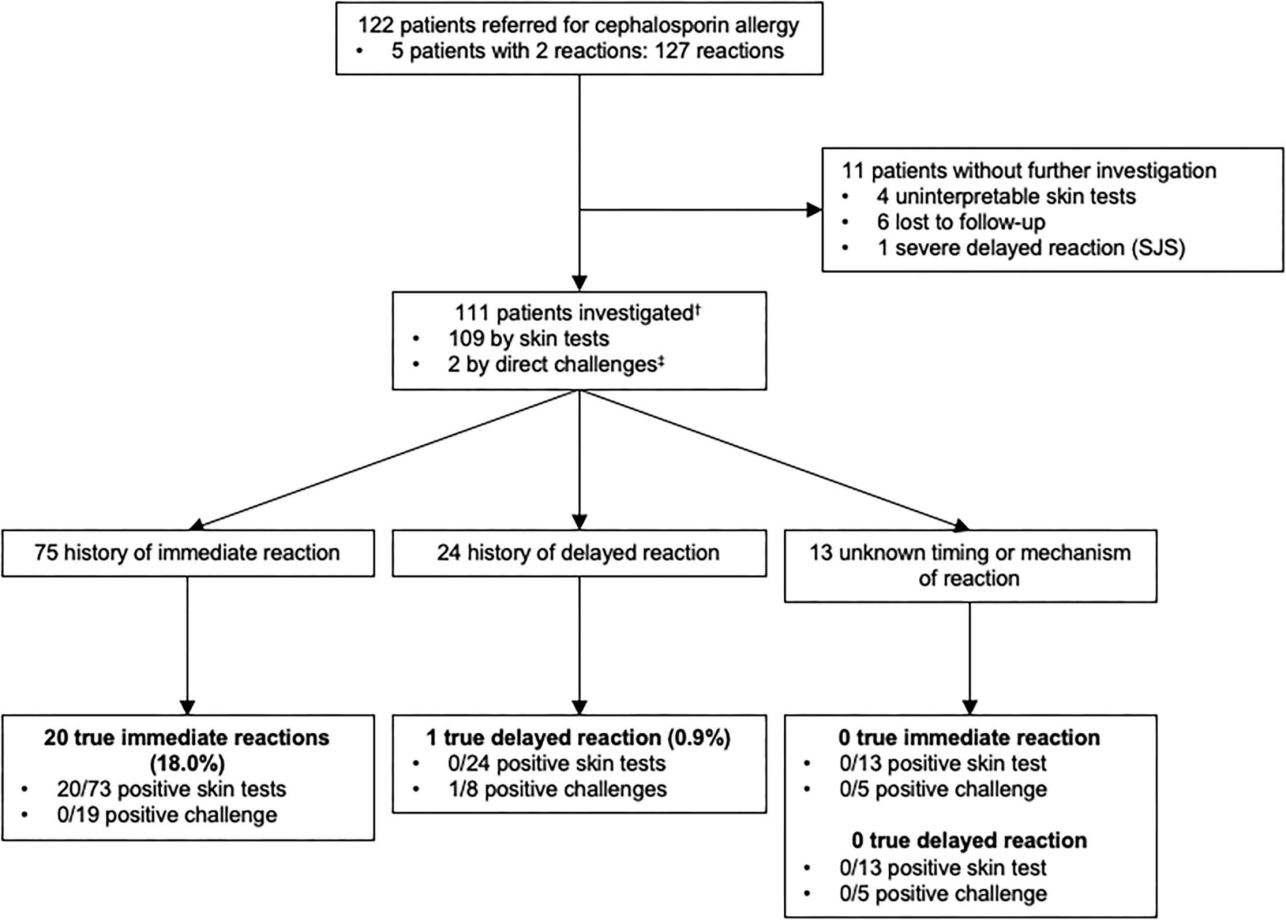
Outcomes of allergy testing in 111 patients with a history of allergy to cephalosporin antibiotics.^†^Among 111 patients who were studied for a history of allergy to cephalosporin antibiotics, some of them had multiple reactions and were included in more than one group. ^‡^2 patients had negative direct drug challenge without prior skin testing (2 direct for a suspicion of an immediate-type reaction). Direct drug challenges were performed on the basis of the physician's assessment because the risk of a reaction was highly unlikely. SJS, Stevens-Johnson syndrome.

Similarly to what was performed for penicillins, we analyzed patients with a history of allergy to cephalosporins (Figure 3). Among 122 patients who reported an allergy to cephalosporins, 111 were investigated. Of note, 1 patient reported 2 reactions to cephalosporins (1 immediate-type reaction and 1 reaction for which no information was available) and was included in the immediate and unknown timing or mechanism of the reaction groups.

Seventy five patients reported symptoms of immediate-type reaction to cephalosporins with a total of 77 reactions. These included 55.8% (*N* = 43) stage IV, 15.6% (*N* = 12) stage III, 24.7% (*N* = 19) stage I–II and 3.9% (*N* = 3) unspecified according to Müller's classification of immediate-type allergic reaction. 18% (20/111) of the patients had a confirmed immediate hypersensitivity to cephalosporins.

Delayed-type reactions to cephalosporins included 24 patients with a total of 26 reactions: 92.3% (*N* = 24) of benign skin rashes and 7.7% (*N* = 2) where the clinical characteristics of the reaction were not available. Only 0.9% (*N* = 1) had a confirmed delayed hypersensitivity to cephalosporins.

Moreover, in 13 patients, there was insufficient information in the medical file to identify the type of the reaction.

In conclusion, the prevalence of confirmed cephalosporins allergies was 18.9% (21/111) overall, 18% (20/111) for immediate-type and 0.9% (1/111) for delayed-type reactions.

### Performance of Skin Testing

Two hundred and twenty five patients had both skin tests and drug challenges. For penicillin skin tests, the sensitivity and the NPV were 90.7% (39/43, [95% CI 82.0–99.4]) and 96.3% (104/108, [95% CI 92.7–99.9]), respectively, for immediate-type reactions, and 84.2% (16/19, [95% CI 67.8–100]) and 91.9% (34/37, [95% CI 83.1–100]), respectively for delayed-type reactions. For cephalosporin antibiotics, we found a NPV of 100% (17/17, [95% CI 100–100]) and 87.5% (7/8, [95% CI 64.6–100]) for immediate- and delayed-type reactions, respectively. Due to the small number of patients with proven allergy to cephalosporin antibiotics, we were unable to accurately determine the sensitivity of the skin tests.

### Safety of Skin Testing and Drug Challenges

Safety of skin testing and drug challenges are detailed in Table 3. Of the 459 patients who underwent skin testing, 3 (0.7%) developed systemic adverse immediate-type reactions.

**Table 3:** Safety of skin tests in 459 patients and in 288 drug challenges.

**Positive Skin tests**		**Positive Challenges**	
**Beta-lactam** ***n*** **(%)**	**3/459**^†^ **(0.7)**	**Beta-lactam** ***n*** **(%)**	**9/288** ^§^ **(3.1)**
Prick	1 (0.2)	Immediate	4 (1.4)
IDR	3 (0.7)	Delayed	5 (1.7)
**Penicillin**	**2/423**^‡^ **(0.5)**	**Penicillin**	**8/228 (3.5)**
Prick	1 (0.2)	Immediate	4 (1.7)
Generalised urticaria^‡^	1 (0.2)	Grade I (hives)	2 (0.9)
IDR	2 (0.5)	Grade III (bronchospasm)	1 (0.4)
Generalised urticaria^‡^	1 (0.2)	Grade IV (shock)	1 (0.4)
Bronchospasm	1 (0.2)	Delayed	4 (1.7)
		Cutaneous reaction^¶^	3 (1.3)
		Hypersensitivity of type III^¶¶^	1 (0.4)
**Cephalosporin**	**1/295 (0.3)**	**Cephalosporin**	**1/60 (1.7)**
Prick	0	Hypersensitivity of type III^¶¶¶^	1 (1.7)
IDR	1 (0.3)		
Malaise and hypotension	1 (0.3)		

† *459 patients underwent skin tests for beta-lactam antibiotics, several of them were tested for different subclasses (penicillins and/or cephalosporins)*.

‡ *1 patient developed generalized skin hives after prick and intradermal skin tests to amoxicillin/clavulanate*.

§ *288 challenges were performed: 12 negative direct drug challenges (10 with a penicillin and 2 with a cephalosporin) without prior skin testing and 276 challenges were preceded by negative skin testing. The decision to proceed with a direct drug challenge (i.e., without prior skin testing) was based on the physician's assessment because the risk of a reaction was highly unlikely*.

¶ *2 maculopapular eruptions occurred 6 and 12 h after a single dose-challenge, and one occurred on day 2 of a 3-day challenge*

¶¶ *This patient developed incomplete serum sickness with generalized urticarial 10 h after a single-dose challenge*.

¶¶¶ *This patient developed arthromyalgia with cutaneous maculopapular eruption 6 h after a single-dose challenge*.

Overall, 288 challenges were performed and 9 (3.1%) patients developed an adverse reaction. 4 (1.4%) immediate-type reactions and 5 (1.7%) delayed-type reactions were reported.

## Discussion

In this study, we evaluated 576 patients who were referred to our university hospital allergy clinic for a history of allergy to one of the beta-lactam antibiotics. Overall, 678 reactions were described.

Since immediate- and delayed-type allergies have different pathophysiological mechanisms, patients were separated into distinct groups according to the clinical history of their reactions: immediate (309/678 = 45.6%), delayed (172/678 = 25.4%) or unknown timing or mechanism of the reaction (138/678 = 20.4%). Each group had similar demographic characteristics. Interestingly, there is a significant preponderance of women in each patient group, with a female to male ratio of ~2:1. This aspect is in accordance with previously published studies (9, 10). Review of the clinical history indicated that the most commonly suspected drugs were penicillins (544/678 = 80.2%) followed by cephalosporins (127/678 = 18.7%). This distribution primarily reflects the frequency of beta-lactam subclasses prescription.

Our main aim was to assess the prevalence of confirmed beta-lactam allergies in the study population. Considering global beta-lactam allergy, allergy was confirmed in 13.4% of the patients with a history of immediate-type reaction, while delayed-type allergy was confirmed in only 4.9% of the patients. Overall, the prevalence of confirmed allergies to beta-lactam antibiotics based on positive skin tests and drug challenges was 18.3%.

Deeper examination of subclasses of beta-lactam antibiotics highlighted that confirmed penicillins immediate-type allergies occurred in 10.9% of the patients and confirmed delayed-type allergies in 5.6%. The results, particularly for immediate-type allergy, closely match previous published data, as most of the studies show that around 90% of the patients who are labeled with penicillins allergy tolerate this drug (5, 11, 12).

Regarding cephalosporins allergy, 18% had a confirmed immediate-type allergy, while only 0.9% of patients were identified with an actual delayed-type allergy. The data available on the epidemiology of cephalosporins allergy are more limited than those on penicillins allergy (13–17). A recent large French retrospective study found that allergy to cephalosporins was proven in 22.3% of the patients and the majority of these reactions were immediate-type (18). According to our data, patients who report a history of allergy to cephalosporins are more likely to be allergic than patients who report an allergy to penicillins (overall 18.9% vs. 16.4% of patients). Furthermore, we confirm that a delayed-type allergy to cephalosporins is a rare condition, as already shown by others (13, 18).

We found that NPV of skin tests for penicillin antibiotics were 96.3% and 91.9% for immediate- and delayed-type reactions, respectively, meaning that patients with negative skin tests will unlikely present an allergic reaction on drug challenge. Sensitivity of skin tests for penicillins was 90.7% for immediate-type reactions and 84.2% for delayed-type ones. These values are similar to data previously reported in retrospective and prospective studies (19, 20). For immediate- and delayed-type reactions to cephalosporins, we determined a NPV of skin tests of 100 and 87.5%, respectively. These results are based on 17 patients for immediate-type, and 8 patients for delayed-type reactions, which likely overestimate these values compared to recent larger studies (18).

Safety of skin tests showed that only 0.7% of the patients presented a systemic allergic reaction. Importantly, only one patient developed a malaise with a transient hypotension. All patients requested antihistamine treatment and for two of them (one for bronchospasm and one for generalized skin hives) the inhalation of a bronchodilator. Beta-lactam skin testing is regarded to be a safe procedure if performed by a trained medical team who is prepared to treat anaphylaxis (5, 11, 21). Assessment of the safety of beta-lactam drug challenges indicated a rate of systemic allergic reactions of 3.1%. These data are similar to previously published results (11, 12, 22–28). Careful examination of these systemic allergic responses showed that most of the reactions were benign skin rashes (hives or maculopapular skin eruptions) but two anaphylaxes were reported and, interestingly, two type-III reactions (purpuric skin rash, arthralgia and fever). These retrospective data emphasize that beta-lactam drug challenge is globally a safe procedure, which should take place in a medical care structure prepared to recognize and treat drug hypersensitivity.

The limitations of this research include the low number of patients in some subgroups. Accordingly, results of NPV for cephalosporins are based on only 17 patients for immediate-type reactions, and 8 patients for delayed-type reactions, which likely overestimate these values compared to recent larger studies (18).

Of note, there are no clear guidelines for defining the duration of the drug challenge for delayed-type reaction. Therefore, in this real-life study, delayed challenges were performed either with a single drug dose (*N* = 36) or with consecutive administration of the drug for 3 days (*N* = 17). Our analysis of the NPV and skin tests sensitivity for delayed-type reactions did not take this variability into account. Therefore, our results were influenced by the duration of the challenges, and the conclusions of these results should be taken with some caution.

In conclusion, in this study we examined patients with a history of allergy to beta-lactam antibiotics under real-life conditions. We have shown that a complete allergy workup, including careful evaluation of the medical history, skin tests and drug challenge, helps to delabel patients who are not truly allergic, and who represent the vast majority of patients included in the present study. This approach contributes to save second line antibiotics that are associated with more side effects and increased antimicrobial resistance. In addition, a standardized allergy workup is a safe procedure if performed by a trained medical team ready to identify and treat allergy and anaphylaxis.

## Data Availability Statement

The raw data supporting the conclusions of this article will be made available by the authors, without undue reservation.

## Ethics Statement

The studies involving human participants were reviewed and approved by Ethics Committee of the Canton of Vaud, Switzerland (CER-VD 2019-00337). The ethics committee waived the requirement of written informed consent for participation.

## Author Contributions

DC: conception and design of the study. SI: acquisition and analysis of the data. SI, LS, LM, YM, CR, GB, and DC: interpretation of data. SI and DC: drafting the manuscript. LS, LM, YM, CR, and GB: revising the manuscript. SI, LS, LM, YM, CR, and DC: final approval of the version to be published and agreement to be accountable for all aspects of the work in ensuring that questions related to the accuracy or integrity of any part of the work are appropriately investigated and resolved. All authors contributed to the article and approved the submitted version.

## Funding

Open access funding provided by University of Lausanne.

## Conflict of Interest

The authors declare that the research was conducted in the absence of any commercial or financial relationships that could be construed as a potential conflict of interest.

## Publisher's Note

All claims expressed in this article are solely those of the authors and do not necessarily represent those of their affiliated organizations, or those of the publisher, the editors and the reviewers. Any product that may be evaluated in this article, or claim that may be made by its manufacturer, is not guaranteed or endorsed by the publisher.
