# Supplementary material for: Management of Beta-Lactam Antibiotics Allergy: A Real-Life Study
Source: Front Allergy. 2022 Apr 8;3:853587. doi: 10.3389/falgy.2022.853587 (PMC9234877; doi:10.3389/falgy.2022.853587)
Supplement: Supplementary file 1 [file Data_Sheet_1.PDF]

Supplementary Material

Supplementary Table 1. Skin tests evaluation

Skin test concentration
**Amoxicillin, amoxicillin/clavulanate and piperacillin**
Prick-test: 25 mg/ml.
Intradermal reaction (IDR): 0.25 mg/ml, IDR 2.5 mg/ml and IDR 25 mg/ml.
**Benzylpenicillin**
Prick-test: 10'000 UI/ml.
IDR 100 UI/ml and IDR 10'000 UI/ml.
**Cephalosporin**
Prick-test 2 mg/ml.
**PPL (penicilloyl-polylysine) and MDM (minor determinant mixture)**
Prick-tests: 1/1
IDR: 1/1

The aforementioned concentrations were used for skin test evaluation. Skin tests were read at 20 min for immediate-type reactions and/or at 48h and 96h for delayed-type reactions.

Supplementary Table 2.

	Immediate		Delayed	
	Prick	IDR	Prick	IDR
**Skin test reagent**				
PPL	3/322	11/325	0/108	0/112
MDM	2/332	8/333	0/111	0/115
Benzylpenicillin	3/351	15/368	0/134	3/156
Amoxicillin	5/172	11/178	0/58	9/70
Amoxicillin/clavulanate	6/244	17/251	0/99	8/116
Flucloxacillin	0/7	0/7	0/4	0/4
Piperacilin/tazobactam	0/30	3/34	0/10	3/14
Cefuroxime	5/245	18/247	0/94	1/103
Ceftriaxone	0/48	3/50	0/13	0/16
Cefazolin	0/15	1/16	0/3	0/3
Ceftazidime	0/2	0/2	0/0	0/0
Cefepime	0/11	0/13	0/3	0/9
Cefixime	0/1	0/1	0/0	0/0
Cefpodoxime	0/1	0/1	0/0	0/0

A. Summary of skin testing for each specific beta-lactam antibiotics. Data are presented as: number of positive skin test/number of skin test performed.

**Drug challenge**	**Immediate**	**Delayed**
**Penicillins**	**4/228**	**4/228**
Phenoxymethylpenicillin	0/8	0/8
Amoxicillin	0/40	2/40
Amoxicillin/clavulanate	4/176	2/176
Flucloxacillin	0/2	0/2
Piperacilin/tazobactam	0/2	0/2
**Cephalosporins**	**0/60**	**1/60**
Cefuroxime	0/46	0/46
Ceftriaxone	0/9	1/9
Cefazolin	0/2	0/2
Cefpodoxime	0/3	0/3

B. Summary of drug challenges for each specific beta-lactam antibiotics. Data are presented as: number of positive drug challenge/number of drug challenge performed.
